# PDBe: enhanced structural data exploration to facilitate discovery

**DOI:** 10.1093/nar/gkaf1120

**Published:** 2025-12-10

**Authors:** Marcelo Querino Lima Afonso, Ivanna Pidruchna, Sreenath Nair, Adam Midlik, Dare Lawal, Melanie Vollmar, Sri Devan Appasamy, Preeti Choudhary, Ibrahim Roshan Kunnakkattu, Damian Bertoni, Cristian A Escobar, Balakumaran Balasubramaniyan, Romana Gaborova, Grisell Díaz Leines, Deborah Harrus, Deepti Gupta, Genevieve L Evans, Urmila Paramval, Paulyna Magana, Ahsan Tanweer, Mihai Todor, Christopher J Thorpe, Maxim Tsenkov, Sudakshina Ganguly, Joseph Ellaway, Weslley Morellato Bueno, Adam Bellaiche, David Sehnal, Radka Svobodová, Jennifer R Fleming, Sameer Velankar

**Affiliations:** European Molecular Biology Laboratory, European Bioinformatics Institute, Hinxton, CB10 1SD, United Kingdom; European Molecular Biology Laboratory, European Bioinformatics Institute, Hinxton, CB10 1SD, United Kingdom; European Molecular Biology Laboratory, European Bioinformatics Institute, Hinxton, CB10 1SD, United Kingdom; European Molecular Biology Laboratory, European Bioinformatics Institute, Hinxton, CB10 1SD, United Kingdom; European Molecular Biology Laboratory, European Bioinformatics Institute, Hinxton, CB10 1SD, United Kingdom; European Molecular Biology Laboratory, European Bioinformatics Institute, Hinxton, CB10 1SD, United Kingdom; European Molecular Biology Laboratory, European Bioinformatics Institute, Hinxton, CB10 1SD, United Kingdom; European Molecular Biology Laboratory, European Bioinformatics Institute, Hinxton, CB10 1SD, United Kingdom; European Molecular Biology Laboratory, European Bioinformatics Institute, Hinxton, CB10 1SD, United Kingdom; European Molecular Biology Laboratory, European Bioinformatics Institute, Hinxton, CB10 1SD, United Kingdom; European Molecular Biology Laboratory, European Bioinformatics Institute, Hinxton, CB10 1SD, United Kingdom; European Molecular Biology Laboratory, European Bioinformatics Institute, Hinxton, CB10 1SD, United Kingdom; CEITEC – Central European Institute of Technology, Masaryk University, Brno625 00, Czech Republic; European Molecular Biology Laboratory, European Bioinformatics Institute, Hinxton, CB10 1SD, United Kingdom; European Molecular Biology Laboratory, European Bioinformatics Institute, Hinxton, CB10 1SD, United Kingdom; European Molecular Biology Laboratory, European Bioinformatics Institute, Hinxton, CB10 1SD, United Kingdom; European Molecular Biology Laboratory, European Bioinformatics Institute, Hinxton, CB10 1SD, United Kingdom; European Molecular Biology Laboratory, European Bioinformatics Institute, Hinxton, CB10 1SD, United Kingdom; European Molecular Biology Laboratory, European Bioinformatics Institute, Hinxton, CB10 1SD, United Kingdom; European Molecular Biology Laboratory, European Bioinformatics Institute, Hinxton, CB10 1SD, United Kingdom; European Molecular Biology Laboratory, European Bioinformatics Institute, Hinxton, CB10 1SD, United Kingdom; European Molecular Biology Laboratory, European Bioinformatics Institute, Hinxton, CB10 1SD, United Kingdom; European Molecular Biology Laboratory, European Bioinformatics Institute, Hinxton, CB10 1SD, United Kingdom; European Molecular Biology Laboratory, European Bioinformatics Institute, Hinxton, CB10 1SD, United Kingdom; European Molecular Biology Laboratory, European Bioinformatics Institute, Hinxton, CB10 1SD, United Kingdom; European Molecular Biology Laboratory, European Bioinformatics Institute, Hinxton, CB10 1SD, United Kingdom; European Molecular Biology Laboratory, European Bioinformatics Institute, Hinxton, CB10 1SD, United Kingdom; National Centre for Biomolecular Research, Faculty of Science, Masaryk University, Brno625 00, Czech Republic; CEITEC – Central European Institute of Technology, Masaryk University, Brno625 00, Czech Republic; European Molecular Biology Laboratory, European Bioinformatics Institute, Hinxton, CB10 1SD, United Kingdom; European Molecular Biology Laboratory, European Bioinformatics Institute, Hinxton, CB10 1SD, United Kingdom

## Abstract

Protein Data Bank in Europe (PDBe) is a founding member of the worldwide Protein Data Bank (wwPDB), delivering open access to experimentally determined macromolecular structures. PDBe also delivers enriched annotations contributed by the PDBe-Knowledge Base (PDBe-KB) consortium. The macromolecular entry pages are the primary interface for millions of users who explore experimental structure data. Here, we describe the redesign of the PDBe entry pages that organize content into logical views, thereby improving usability and facilitating the use of structure models, driving fundamental and applied research, and supporting education. The new design introduces several enhancements, including integrated and central 3D visualization, sequence feature exploration, standardized molecular scenes, AI-driven residue-level annotations from text mining of the literature, and streamlined annotation access via refactored APIs. Importantly, researchers can now upload their own residue-level features and visualize them directly in structural context, displayed alongside PDBe-KB annotations. This functionality enables scientists to interpret unpublished or private data in relation to high-quality structural information, lowering barriers for those without prior expertise in structural biology. Together, these updates create a more accessible, flexible, and scalable framework for interacting with structural data, expanding the resource’s value to both domain specialists and the wider life sciences community.

## Graphical Abstract

**Graphical Abstract:**
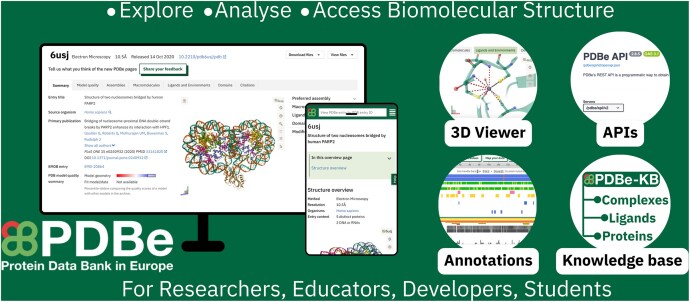

## Introduction

The structural biology landscape has changed dramatically in recent years. Advances in cryo-electron microscopy enable routine determination of large macromolecular assemblies [1], while AI-based approaches such as AlphaFold have accelerated experimental structure determination and extended structural coverage to entire proteomes [2–4]. Yet the sheer scale and complexity of these data can present barriers: without clear, intuitive entry points, researchers may struggle to extract biological meaning, limiting the broader impact of structural advances.

The Protein Data Bank in Europe (PDBe) is a founding member of the Worldwide Protein Data Bank (wwPDB) [5], providing open access to experimentally determined biomacromolecular structures and their metadata. Building on this foundation, the PDBe-Knowledge Base (PDBe-KB) [6] enriches structural entries with functional annotations from expert community resources, linking molecular structure to biological context. Together, these resources form an integrated platform that makes structural data not only openly accessible but also interpretable and reusable for the widest possible community, from structural specialists to researchers in genomics, pharmacology, and systems biology. The redesign of PDBe entry pages directly addresses the challenge of transforming structural complexity into usable biological insight. By improving clarity, navigation, and contextualization, the new interface provides a bridge between raw experimental information and life science applications, enabling researchers across disciplines to integrate structural perspectives into their studies and amplify the impact of the wealth of available structural data.

Recent PDBe initiatives have focused on enriching the interpretability of structural data. Conformational states identified across the archive are displayed on PDBe-KB protein pages [7], ligand analysis and classification tools are integrated into PDBe-KB ligand pages [8], and macromolecular assembly annotations, including persistent identifiers [9] and biological context, will soon be available through dedicated PDBe-KB complex pages. Building on this foundation, and linking to these resources, the redesigned PDBe entry pages adopt a modular tab-based layout that improves clarity and navigation while retaining the full details of structural experiments. Enhancements include: Mol*-based 3D visualization with MolViewSpec-defined focus views [10]; synchronized sequence annotations through Nightingale tracks [11]; revised model quality reporting; AI-driven literature mining of residue-level annotations [12]; and a publication figure viewer. Together, these developments shift PDBe from being solely a repository of structural experiments towards a comprehensive knowledge resource that connects molecular structure with biological function and context, supporting both expert and non-expert users while adhering to FAIR (Findable, Accessible, Interoperable, Reusable) principles.

## Data infrastructure and programmatic access

PDBe-KB integrates macromolecular structure data and annotations using PDBe’s graph-based data model, which has been in active development since 2019 [13]. Implemented in Neo4j, this model connects proteins, ligands, domains, assemblies, and external references such as UniProt [14] and SIFTS [15] cross-references to Pfam [16], InterPro [17], SCOP [18], and CATH [19], creating a rich knowledge graph that supports complex biological queries and data discovery. As part of the portal redesign, we have undertaken a significant upgrade to unify our Application Programming Interface (API) endpoints, consolidating previously separate API services into a streamlined infrastructure. APIs are a standard way for different software systems to exchange information, enabling more reliable and consistent access to our data. This restructuring consolidates the entry-based APIs and aggregated APIs into a single, comprehensive framework that serves both the public web interface and programmatic access needs. In practical terms, this restructuring means that information about entries, assemblies, ligands, and annotations can now be retrieved through a single, consistent system rather than several separate services. This unified approach makes it easier for both the PDBe website and external users to access the same up-to-date data, reducing redundancy and improving performance. New API endpoints are added as needed to support emerging data types and services. For more details and the most up-to-date information, please refer to our documentation at www.ebi.ac.uk/pdbe/pdbe-rest-api. This infrastructure delivers enhanced performance with improved response times, reduced latency and a consistent approach to identifiers, annotations, and relationships across diverse data types.

Macromolecular structure and annotation data are now organized hierarchically into ligands, domains, individual chains, and assemblies and are dynamically generated from unified API endpoints drawing on both graph and relational database backends. The new infrastructure features an expanded set of endpoints specifically designed for macromolecular assemblies, entry, and ligand data retrieval, emphasizing standard data formats, comprehensive OpenAPI specifications (https://swagger.io/specification/), and efficient bulk access capabilities to support high-throughput research workflows. This graph-based design enables users to begin with one entity, such as a ligand, and traverse across related entities—for example, to its binding partners, associated domains, or assemblies—while retaining links to experimental details and model quality. In this way, the new infrastructure facilitates exploration of structural data in its biological context and establishes a robust foundation for future extensions, including predicted assemblies and domain-level views.

## New web features and page types

The portal redesign introduces a set of new and restructured page types that improve accessibility and visual clarity, and provide a consistent user experience. To support both interactive use and programmatic access, all web data displayed on these pages is also retrievable via the unified PDBe API.

Entry Pages, which remain the primary access point for individual structural experiments, now adopt a modular tab-based design tailored to diverse users, including structural biologists, computational and life science researchers, educators, and clinicians. Core structural components—macromolecules, ligands, domains, and macromolecular assemblies—are organized into dedicated tabs, with integrated 3D visualization supporting predefined focus views (such as assemblies, ligands, domains, and modified residues). A collapsible summary panel provides rapid access to key features.

**Figure 1.**
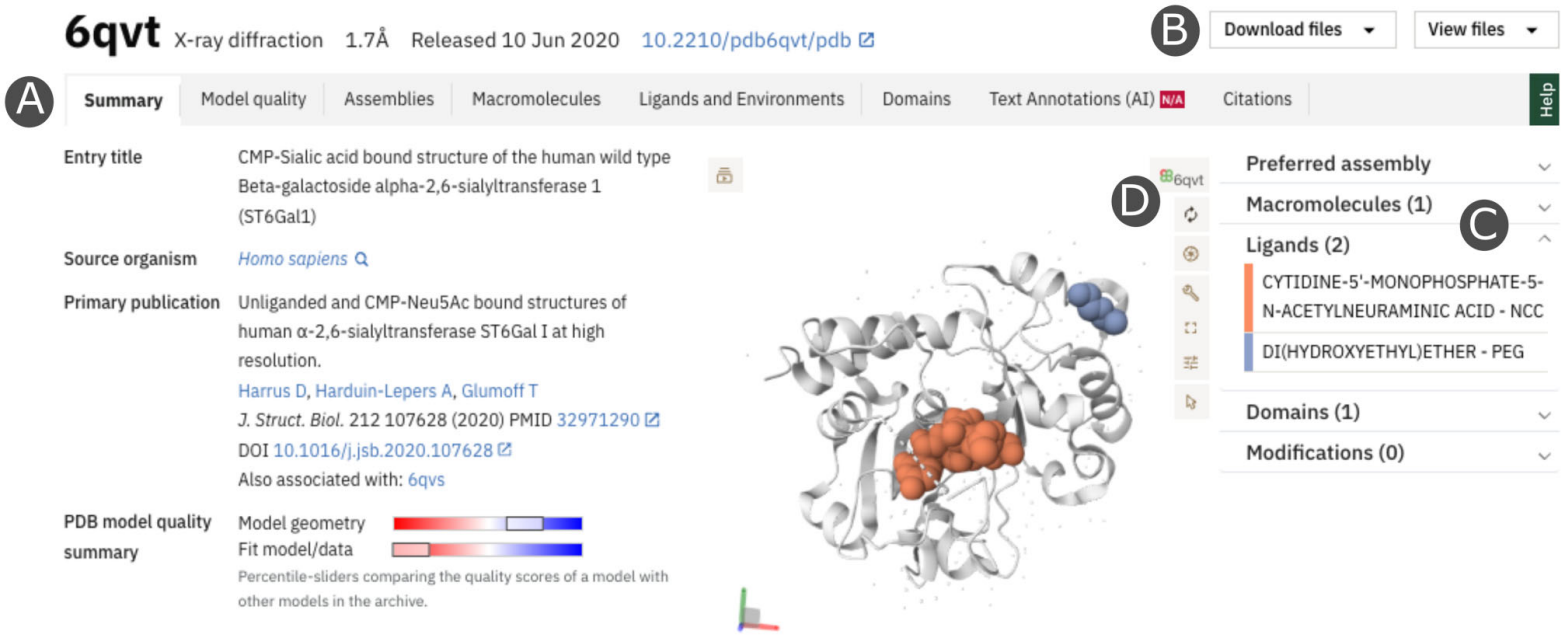
PDBe Entry Page. (**A**) New tab design. (**B**) Downloads have been grouped and now also have a search function. (**C**) Preset views can be selected from the summary accordion. (**D**) Mol* controls, allowing snapshots, expansion to full-screen mode, and other functions (see the REF for more features).

Notably, annotation tabs enable researchers to map their own residues or features of interest onto the 3D structure and display these alongside the precomputed PDBe-KB annotations (Fig. 1). This functionality helps researchers interpret their own private or unpublished datasets in a structural context and supports hypothesis generation. Crucially, the data are never uploaded or shared as everything remains entirely within the user’s browser. By lowering barriers for those unfamiliar with macromolecular graphic viewers, it also broadens the use of structural data by clinicians, educators, and researchers in related fields (Fig. 1).

**Figure 2.**
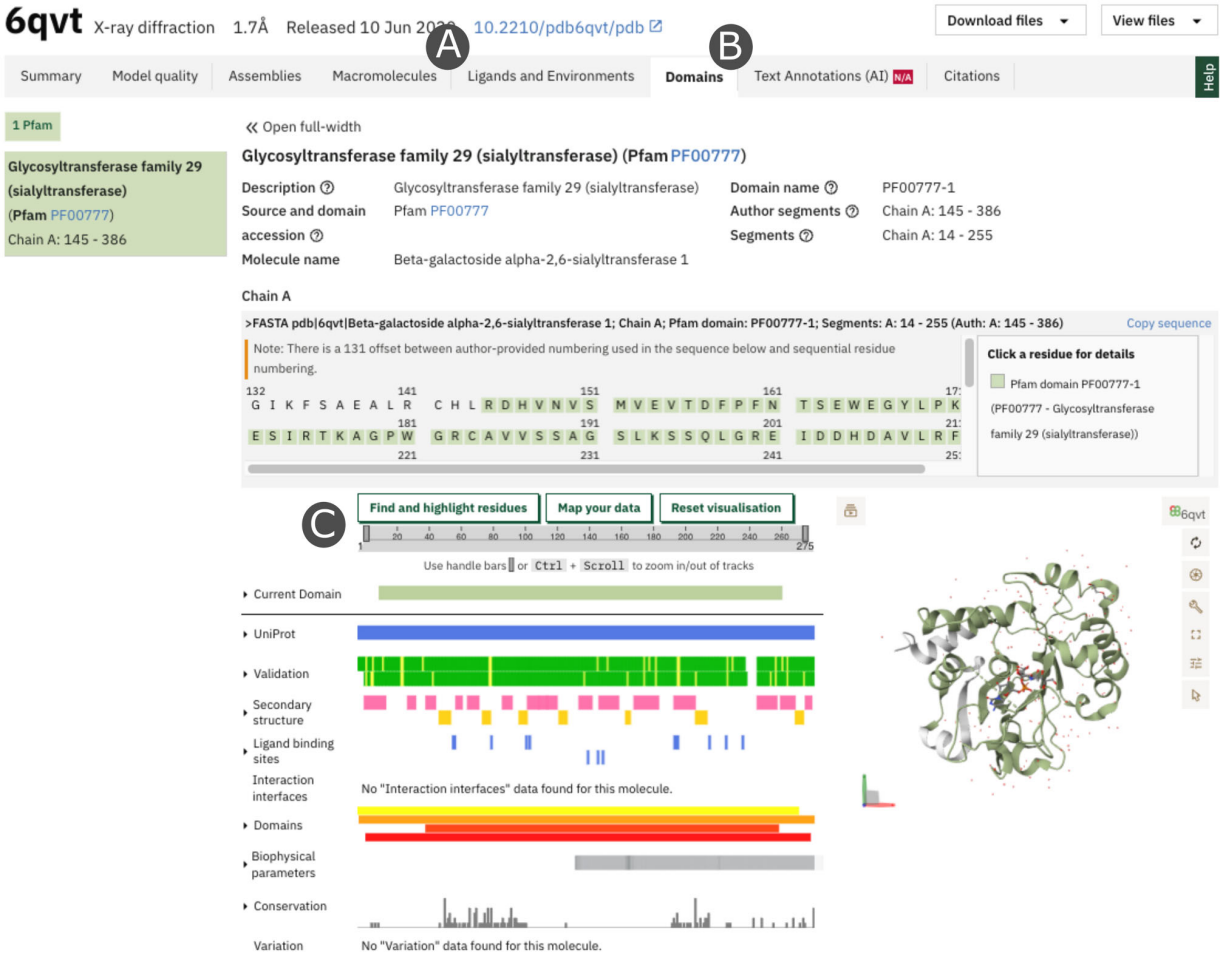
Customizable annotation is available on the Macromolecules (**A**) and Domains (**B**) tabs, allowing users to contextualize their own data within the precalculated PDBe-KB annotations. For example, a cancer researcher can upload mutation sites into ProtVista (**C**) and instantly see them in 3D context with known ligand-binding residues, enabling rapid functional interpretation without prior training in structure visualization.

The entire portal employs a unified 3D visualization framework via Mol* [10]. Visualization states are defined through MolViewSpec (MVS) [20, 21], a declarative schema that supports interoperability between tools and enables consistent and reproducible rendering of molecular scenes across PDBe pages. This framework synchronizes with 2D sequence tracks in ProtVista [11], expediting integrated exploration of sequence and structural annotations (Fig. 2). Users can now also upload their own residue selections into ProtVista, which are then synchronized with Mol* and the sequence viewer. This allows custom annotations, such as functional sites, mutations, or experimental features, to be directly mapped in the context of pre-computed PDBe-KB annotations, supporting comparative analysis and interpretation (Fig. 2C). The sequence viewer also handles multiple numbering schemes introduced by differences between UniProt sequence numbers and PDB author numbering, as well as non-trivial residue indexing, ensuring ease of alignment between uploaded data, sequence tracks, and structural views.

Model quality summaries and validation metrics are presented with enhanced contextual information and improved annotation tools, facilitating assessment of the structural model. For crystallographic entries, experimental parameters and refinement statistics are displayed in an expanded tabular format, providing a clearer context for data interpretation.

Another significant addition is the integration of AI-derived annotations, which provide residue-level insights extracted from the scientific literature representing the primary citation for an entry. Mentions of specific residues are identified through automated text mining in collaboration with IUCr journals and linked to corresponding PDB entries and UniProt accessions [22]. This feature provides direct access to relevant biological context via PDBe API download and visualization on our webpages, complementing structural data. These annotations are visualized in ProtVista and Mol*, complementing both precomputed and user-provided annotations and allowing the user to fully explore the biology of the PDB entry in an unprecedented way.

The citation section has been updated to display both primary publications and subsequent entry data usage patterns, while a figure viewer presents selected images from open-access articles to contextualize structures within their experimental framework.

We are now working towards a consistent interface design that spans individual entry and links to the aggregated PDBe-KB pages for proteins (www.ebi.ac.uk/pdbe/pdbe-kb/proteins/Q14676), ligands (www.ebi.ac.uk/pdbe-srv/pdbechem/chemicalCompound/show/GOL), and, most recently, macromolecular assemblies (www.ebi.ac.uk/pdbe/pdbe-kb/complexes/1a00), facilitating navigation across related data types. The portal is responsive and cross-platform optimized, ensuring core functionality and visualization are accessible on both desktop and mobile devices.

The redesign incorporated feedback from the structural biology and broader research communities through systematic surveys, interviews, and usability testing. Community input identified priorities, including navigation clarity, visual consistency, and better support for multi-omics integration. These requirements shaped the redesign, and ongoing engagement continues to guide development as structural data are applied across increasingly diverse research contexts. This consultation has enabled the evolution of the PDBe portal into a knowledge resource, with modular entry pages, unified infrastructure, and centralized 3D visualization that support both pre-computed and user-provided annotations. The pages are continually optimized to ensure accessibility and support research and education for users with limited bandwidth settings or computing resources. Looking ahead, PDBe will continue to expand annotation coverage by strengthening community links and adapting to emerging trends such as assemblies, multi-omics integration, AI-driven annotation, and interoperability between experimental and predicted 3D-structure resources, including the AlphaFold Protein Structure Database. In this way, the portal will continue to be a forward-looking platform that empowers the use of 3D-structure data to support discovery, innovation, and education.

## Data Availability

The new PDBe entry pages are available at www.ebi.ac.uk/pdbe/. All data are freely accessible under Creative Commons Zero (CC0) www.ebi.ac.uk/pdbe/about/public-data-access-statement. The unified PDBe API is documented at www.ebi.ac.uk/pdbe/pdbe-rest-api with OpenAPI 3.0 specifications available. Source code for key visualisation components can be found in https://github.com/PDBeurope/ and https://github.com/molstar/pdbe-molstar. Educational materials, including tutorials and webinars, are provided at https://www.ebi.ac.uk/training/online/courses/exploring-pdb-entry/. User support is available through pdbehelp@ebi.ac.uk.
